# Do non-elite older runners slow down more than younger runners in a 100 km ultra-marathon?

**DOI:** 10.1186/2052-1847-7-1

**Published:** 2015-01-09

**Authors:** Christoph A Rüst, Thomas Rosemann, Matthias A Zingg, Beat Knechtle

**Affiliations:** Institute of Primary Care, University of Zurich, Zurich, Switzerland; Gesundheitszentrum St. Gallen, Vadianstrasse 26, 9001 St. Gallen, Switzerland

**Keywords:** Running, Men, Long-distance, Master athlete

## Abstract

**Background:**

This study investigated changes in normalised running speed as a proxy for effort distribution over segments in male elite and age group 100 km ultra-marathoners with the assumption that older runners would slow down more than younger runners.

**Methods:**

The annual ten fastest finishers (*i.e.* elite and age group runners) competing between 2000 and 2009 in the ‘100 km Lauf Biel’ were identified. Normalised average running speed (*i.e.* relative to segment 1 of the race corrected for gradient) was analysed as a proxy for pacing in elite and age group finishers. For each year, the ratio of the running speed from the final to the first segment for each age cohort was determined. These ratios were combined across years with the assumption that there were no ‘extreme’ wind events etc. which may have impacted the final relative to the first segment across years. The ratios between the age cohorts were compared using one-way ANOVA and Tukey’s post-hoc test. The ratios between elite and age group runners were investigated using one-way ANOVA with Dunnett’s multiple comparison post-hoc tests. The trend across age groups was investigated using simple regression analysis with age as the dependent variable.

**Results:**

Normalised average running speed was different between age group 18–24 years and age groups 25–29, 30–34, 35–39, 40–44, 45–49, 50–54, 55–59 and 65–69 years. Regression analysis showed no trend across age groups (r^2^ = 0.003, *p* > 0.05).

**Conclusion:**

To summarize, (*i*) athletes in age group 18–24 years were slower than athletes in most other age groups and (*ii*) there was no trend of slowing down for older athletes.

## Background

Ultra-marathon running involves any race distance longer than the classical marathon distance of 42.195 km [1]. Among the different race distances, the 100 km ultra-marathon distance is highly popular where the number of female and male finishes increased exponentially in the last 50 years [2].

For 100 km ultra-marathon running, age [3] and the origin [2] of the fastest finishers are known. For elite 100 km ultra-marathoners, the pacing strategy has also been investigated [4]. Faster runners started at a faster running speed, finished the race within 15% of their starting speed, and maintained their starting speed for ~50 km before slowing down [4]. Slower runners showed a greater percent decrease in running speed and were unable to maintain their initial pace for as long [4]. Apart from elite 100 km ultra-marathoners, the pacing strategy has recently been investigated for elite 161 km ultra-marathoners [5]. Winners in 161 km ultra-marathons remained relatively close behind the leading runners before taking the lead in the middle half of the race, and then avoided slowing down as much as the other top runners in the latter stages of the race [5].

Age seems to be an important predictor variable in running performance in marathoners [6] and ultra-marathoners [7, 8]. Running performance in male 100 km ultra-marathoners was fastest at the age of ~35 years [3] and decreased after the age of ~45 years [9]. Since running performance in 100 km ultra-marathon running decreased with increasing age, we may assume that older runners (*i.e.* older than ~35-45 years) will slow down faster during a 100 km than younger runners (*i.e.* younger than ~35-45 years). This study investigated changes in normalised running speed (as a proxy for effort distribution) over segments in male elite and age group 100 km ultra-marathoners in the ‘100 km Lauf Biel’ held in Switzerland [10] with the assumption that older runners would slow down more than younger runners [11].

## Methods

### Ethics

The present study was approved by the Institutional Review Board of St. Gallen, Switzerland, with a waiver of the requirement for informed consent given that the study involved the analysis of publicly available data.

### The race

The ‘100 km Lauf Biel’ held in Biel, Switzerland, is one of the most traditional and largest 100 km ultra-marathons in Europe [10]. The race takes place during the night of the first weekend in June. All runners start the race at 10:00 p.m. The race held is held as one large loop of 100 km with a total change in altitude of ~645 m. The organizer provides a total of 17 aid stations offering an abundant variety of food and beverages such as hypotonic sports drinks, tea, soup, caffeinated drinks, water, bananas, oranges, energy bars and bread. The athletes are allowed to be supported by a cyclist for additional food and clothing, if necessary. The fastest finishers complete the race within ~7 h and arrive early in the morning at around sunrise of the following day.

### Data sampling and data analysis

The data set for this study was obtained from the race website of the ‘100 km Lauf Biel’ [10]. In this race, split times at three time stations (TS) at TS1 ‘Oberramsern’ (38 km), TS2 ‘Kirchberg’ (56.1 km) and TS3 ‘Bibern’ (76.7 km) were taken using an electronic chip during the 2000–2009 period. In earlier and later years, split times were taken differently. For each edition, we extracted the ten fastest men overall and the ten fastest male age group finishers for 5-year age groups (*i.e.* 18–24 yrs, 25–29 yrs, 30–34 yrs, 35–39 yrs, 40–44 yrs, 45–49 yrs, 50–54 yrs, 55–59 yrs, 60–64 yrs, and 65–69 yrs). Since too few women competed in this race, we had to limit to male competitors. Normalised average running speed (*i.e.* relative to segment 1 of the race corrected for gradient) was analysed as a proxy for pacing in elite (*i.e.* the annual fastest) and age group finishers. Elite runners (*i.e.* the annual ten fastest) were considered as a kind of ‘gold standard’ of pacing from start to end since we may assume that they have got their pacing right. We obtained for each year the ratio of the running speed from the final to the first segment for elite athletes and each age cohort. We combined these ratios across years with the assumption that there were no ‘extreme’ wind events etc. that may have impacted the final segment relative to the first across years. We plotted then the mean and standard deviations of these ratios on a graphic with age cohort on the X-axis.

### Statistical analysis

The ratios between the age cohorts were compared using measures one-way analysis of variance (ANOVA) and Tukey’s post-hoc test. The ratios between elite runners and age group runners were investigated using one-way ANOVA with Dunnett’s multiple comparison post-hoc tests. The trend across age groups (*i.e.* from elite runners to runners in age group 65–69 years) seemed linear and we conducted a simple regression analysis with age as the dependent variable. Statistical analyses were performed using IBM SPSS Statistics (Version 22, IBM SPSS, Chicago, IL, USA) and GraphPad Prism (Version 6.01, GraphPad Software, La Jolla, CA, USA). Significance was accepted at *p* < 0.05 (two-tailed for *t*-tests). Data are given as mean ± standard deviation (SD).

## Results

**Figure 1:**
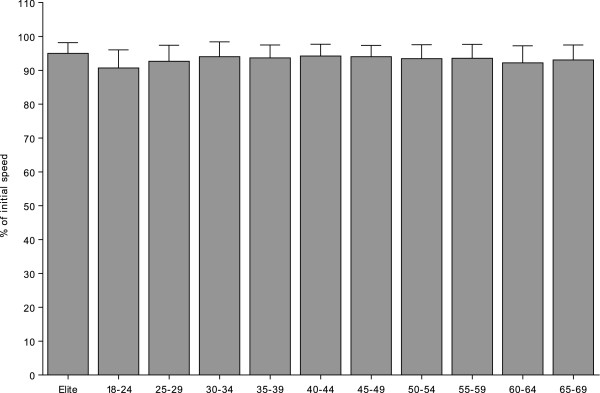
**Comparison of normalised average running speed between elite runners and age group runners.**

Normalised average running speed was different between athletes ranked in age group 18–24 years and athletes ranked in age groups 25–29 years, 30–34 years, 35–39 years, 40–44 years, 45–49 years, 50–54 years, 55–59 years and 65–69 years (Table 1 and Figure 1). Regression analysis showed no trend across age groups (r^2^ = 0.003, *p* > 0.05).

**Table 1:** **Results of the Tukey’s multiple comparisons test**

	Mean 1	Mean 2	Mean diff.	95% CI of diff.	Significance
18-24 *versus* 25-29	90.69	92.65	−1.96	−3.90 to −0.02	*
18-24 *versus* 30-34	90.69	94.03	−3.34	−5.28 to −1.40	****
18-24 *versus* 35-39	90.69	93.67	−2.98	−4.92 to −1.04	****
18-24 *versus* 40-44	90.69	94.20	−3.51	−5.45 to −1.57	****
18-24 *versus* 45-49	90.69	94.03	−3.34	−5.28 to −1.40	****
18-24 *versus* 50-54	90.69	93.46	−2.77	−4.71 to −0.83	***
18-24 *versus* 55-59	90.69	93.57	−2.88	−4.82 to −0.94	***
18-24 *versus* 60-64	90.69	92.19	−1.50	−3.44 to 0.43	ns
18-24 *versus* 65-69	90.69	93.06	−2.37	−4.31 to −0.43	**
25-29 *versus* 30-34	92.65	94.03	−1.37	−3.31 to 0.56	ns
25-29 *versus* 35-39	92.65	93.67	−1.01	−2.95 to 0.92	ns
25-29 *versus* 40-44	92.65	94.20	−1.55	−3.49 to 0.38	ns
25-29 *versus* 45-49	92.65	94.03	−1.37	−3.31 to 0.56	ns
25-29 *versus* 50-54	92.65	93.46	−0.80	−2.74 to 1.13	ns
25-29 *versus* 55-59	92.65	93.57	−0.91	−2.85 to 1.02	ns
25-29 *versus* 60-64	92.65	92.19	0.46	−1.47 to 2.40	ns
25-29 *versus* 65-69	92.65	93.06	−0.40	−2.34 to 1.53	ns
30-34 *versus* 35-39	94.03	93.67	0.35	−1.58 to 2.29	ns
30-34 *versus* 40-44	94.03	94.20	−0.17	−2.11 to 1.76	ns
30-34 *versus* 45-49	94.03	94.03	−0.00	−1.94 to 1.93	ns
30-34 *versus* 50-54	94.03	93.46	0.56	−1.37 to 2.50	ns
30-34 *versus* 55-59	94.03	93.57	0.45	−1.48 to 2.39	ns
30-34 *versus* 60-64	94.03	92.19	1.83	−0.10 to 3.77	ns
30-34 *versus* 65-69	94.03	93.06	0.96	−0.97 to 2.90	ns
35-39 *versus* 40-44	93.67	94.20	−0.53	−2.47 to 1.40	ns
35-39 *versus* 45-49	93.67	94.03	−0.35	−2.29 to 1.58	ns
35-39 *versus* 50-54	93.67	93.46	0.21	−1.73 to 2.15	ns
35-39 *versus* 55-59	93.67	93.57	0.09	−1.84 to 2.03	ns
35-39 *versus* 60-64	93.67	92.19	1.48	−0.45 to 3.42	ns
35-39 *versus* 65-69	93.67	93.06	0.60	−1.33 to 2.54	ns
40-44 *versus* 45-49	94.20	94.03	0.17	−1.76 to 2.11	ns
40-44 *versus* 50-54	94.20	93.46	0.74	−1.19 to 2.68	ns
40-44 *versus* 55-59	94.20	93.57	0.63	−1.30 to 2.57	ns
40-44 *versus* 60-64	94.20	92.19	2.01	0.07 to 3.95	*
40-44 *versus* 65-69	94.20	93.06	1.14	−0.79 to 3.08	ns
45-49 *versus* 50-54	94.03	93.46	0.56	−1.37 to 2.50	ns
45-49 *versus* 55-59	94.03	93.57	0.45	−1.48 to 2.39	ns
45-49 *versus* 60-64	94.03	92.19	1.84	−0.10 to 3.78	ns
45-49 *versus* 65-69	94.03	93.06	0.96	−0.97 to 2.90	ns
50-54 *versus* 55-59	93.46	93.57	−0.11	−2.05 to 1.82	ns
50-54 *versus* 60-64	93.46	92.19	1.27	−0.66 to 3.21	ns
50-54 *versus* 65-69	93.46	93.06	0.39	−1.54 to 2.33	ns
55-59 *versus* 60-64	93.57	92.19	1.38	−0.55 to 3.32	ns
55-59 *versus* 65-69	93.57	93.06	0.51	−1.42 to 2.45	ns
60-64 *versus* 65-69	92.19	93.06	−0.87	−2.81 to 1.06	ns

* = *p* < 0.05, ** = *p* < 0.01, *** = *p* < 0.001, **** = *p* < 0.0001, ns = non significant.

## Discussion

We investigated changes in normalised running speed over segments in male elite and age group 100 km ultra-marathoners in the ‘100 km Lauf Biel’ held in Switzerland and hypothesized that older runners would slow down more than younger runners. The most important findings were (*i*) athletes in age group 18–24 years were slower than athletes in most other age groups and (*ii*) older runners were not slowing down compared to younger runners.

### Athletes in age group 18–24 years were slower than athletes in older age groups

A rather surprising finding was that athletes in age group 18–24 years were slower than athletes in most other age groups. Age has been reported as a major predictor variable in 100 km apart from running speed during training and weekly running kilometers [7]. The age of 18–24 years is not the typical age of successful ultra-marathoners [8]. Ultra-marathoners are typically ~45 years old and male ultra-marathoners achieve their fastest running times at the age of ~ 30–49 years [3, 8]. Cejka et al. [3] investigated the change in 100 km running performance and in the age of peak performance for 100 km ultra-marathoners competing worldwide between 1960 and 2012. Considering the world-wide trend in 100 km ultra-running, the fastest race times are achieved by athletes at the age of ~40 years. Running speed of the top ten athletes increased across calendar years from 8.67 km/h to 15.65 km/h and the age of the top ten athletes increased across calendar years from 29 years to 40 years [3]. The age of the best ultra-marathon performance is also higher in the longest race distances. When athletes competing from 6 hours to 10 days were investigated, the age of the best ultra-marathon performance increased with increasing race duration [12].

Athletes at the age of ~20 years may also have too little experience in ultra-marathon running. Younger ultra-marathoners have not finished as many ultra-marathoners as older ultra-marathoners have [8]. Hoffman and Krishnan [13] investigated the past-year and lifetime exercise patterns of 1,345 current and former ultra-marathoners. Median age at the first ultra-marathon was 36 years, and the median number of years of regular running before the first ultra-marathon was 7 years [13]. Active ultra marathoners had a previous year median running distance of 3,347 km, which was minimally related to age but mostly related to their longest ultra-marathon competition of the year. A recent study investigating ultra-marathoners competing from 6 hours to 10 days showed that the age of peak ultra-marathon performance increased with increasing number of finishes [12].

### Older runners were not slowing down compared to younger runners

We hypothesized that older runners would slow down more than younger runners. However, older runners were not slowing down compared to elite and younger age group runners. Apart from the aspect of experience discussed above, also psychological reasons for decision taking in pacing strategy should also be considered [14–16]. The performance level of the athletes seems the most important reason. Renfree and St Clair Gibson [14] investigated the influence of different performance standards on pacing strategy in elite female marathoners competing in the IAAF Women’s Marathon Championship in 2009. Athletes finishing in the first 25% of all finishers ran the first two 5-km segments at a relatively lower speed than athletes in the second to fourth 25% of all finishers but at a relatively higher sped after km 35. The authors concluded that psychological factors influenced decision making in a major competitive event. Similarly, top runners in the ‘New York City Marathon’ tried to maintain an even pacing profile by avoiding an excessively fast start which might result in a decrease in running speed in the second half of the race [15]. Esteve-Lanao et al. [16] investigated the pacing strategy in World Cross Country Championships between 2007 and 2013. They showed that top ten finishers displayed a more even pacing compared to the other finishers who showed a more positive pacing.

### Limitations

This study is limited since the aspect of environmental factors such as wind has not been included. An actual study reported that headwind was significant factor in running speed variability in the world record marathon runs in 2008 and 2011 [17].

### Practical applications

This analysis shows that athletes in age group 18–24 years were slower than athletes in most other age groups. Younger athletes intending to compete in a 100 km should be very cautious about pacing and are recommended to complete many shorter races in advance.

## Conclusions

In summary, the comparison of normalized average running speed between elite runners and age group runners showed that athletes in age group 18–24 years were slower than athletes in most other age groups. Across age groups, there was no trend of slowing down for older athletes compared to younger athletes.
